# Examination of the Addictive and Behavioral Properties of Fatty Acid-Binding Protein Inhibitor SBFI26

**DOI:** 10.3389/fpsyt.2016.00054

**Published:** 2016-04-05

**Authors:** Panayotis K. Thanos, Brendan H. Clavin, John Hamilton, Joseph R. O’Rourke, Thomas Maher, Christopher Koumas, Erick Miao, Jessenia Lankop, Aya Elhage, Samir Haj-Dahmane, Dale Deutsch, Martin Kaczocha

**Affiliations:** ^1^Behavioral Neuropharmacology and Neuroimaging Laboratory on Addictions, Research Institute on Addictions, University at Buffalo, Buffalo, NY, USA; ^2^Department of Biochemistry, Stony Brook University, Stony Brook, NY, USA; ^3^Department of Anesthesiology, Stony Brook University, Stony Brook, NY, USA

**Keywords:** endocannabinoid, FABP, anandamide, addiction, conditioned place preference, reward, memory, social behavior

## Abstract

The therapeutic properties of cannabinoids have been well demonstrated but are overshadowed by such adverse effects as cognitive and motor dysfunction, as well as their potential for addiction. Recent research on the natural lipid ligands of cannabinoid receptors, also known as endocannabinoids, has shed light on the mechanisms of intracellular transport of the endocannabinoid anandamide by fatty acid-binding proteins (FABPs) and subsequent catabolism by fatty acid amide hydrolase. These findings facilitated the recent development of SBFI26, a pharmacological inhibitor of epidermal- and brain-specific FABP5 and FABP7, which effectively increases anandamide signaling. The goal of this study was to examine this compound for any possible rewarding and addictive properties as well as effects on locomotor activity, working/recognition memory, and propensity for sociability and preference for social novelty (SN) given its recently reported anti-inflammatory and analgesic properties. Male C57BL mice were split into four treatment groups and conditioned with 5.0, 20.0, 40.0 mg/kg SBFI26, or vehicle during a conditioned place preference (CPP) paradigm. Following CPP, mice underwent a battery of behavioral tests [open field, novel object recognition (NOR), social interaction (SI), and SN] paired with acute SBFI26 administration. Results showed that SBFI26 did not produce CPP or conditioned place aversion regardless of dose and did not induce any differences in locomotor and exploratory activity during CPP- or SBFI26-paired open field activity. We also observed no differences between treatment groups in NOR, SI, and SN. In conclusion, as SBFI26 was shown previously by our group to have significant analgesic and anti-inflammatory properties, here we show that it does not pose a risk of dependence or motor and cognitive impairment under the conditions tested.

## Introduction

The endocannabinoid system (ECS), which includes the lipid-derived neurotransmitters arachidonoylethanolamide (anandamide/AEA) and 2-arachidonoyl glycerol (2-AG) (1), has been widely studied (2) and implicated in numerous functions and diseases, including schizophrenia and Parkinson’s (3), pain sensation (4), anxiety (5), learning and memory (6), emotion (7), obesity (8), and drug addiction (9). In the central nervous system, 2-AG and AEA primarily bind to the CB1 receptor (10). This discovery has led to the development of several synthetic compounds that can modify both the anabolism and catabolism of these endogenous signaling molecules.

Arachidonoylethanolamide is hydrolyzed by fatty acid amide hydrolase (FAAH), which is found in abundance throughout the CNS along with CB1 receptors and is the main enzyme responsible for AEA breakdown (11, 12). FAAH inhibitors, such as URB597, significantly elevate AEA levels and have been deemed effective in alleviating depression and anxiety symptoms (13) as well as pain and inflammation (14) in rodents, and at therapeutic doses, they lack the psychomotor deficits associated with direct CB1 receptor agonists (15). However, these compounds lack specificity (16). For example, FAAH is highly expressed in the liver (17), and systemic inhibition is linked with hyperglycemia and insulin resistance (18).

Due to the fact that AEA is a lipid ligand and that its catabolizing enzyme FAAH is localized on the endoplasmic reticulum and mitochondria, it has been deemed likely that a specific mechanism is responsible for the transport of AEA through the cellular cytosol for hydrolysis (19). Fatty acid-binding proteins (FABPs) have been proposed by Kaczocha et al. (20) as the means of transport for AEA. A particular focus has been on FABP3, 5, and 7 that are highly expressed within the brain and may provide a tissue-specific approach to elevating AEA. More recent findings have emerged supporting this claim and the possibility for this transport mechanism as a target for therapeutic modification (21–25). A recently developed compound, SBFI26, inhibits two of the most prevalent FABPs in the brain (FABP5 and FABP7) (21). Kaczocha et al. (26) have shown that SBFI26 inhibits visceral, inflammatory, and neuropathic pain by increasing AEA levels in rodent brain. FABP3 (heart-FABP) was not targeted (although it is also abundant in the CNS), because its deletion has been shown to induce age-related cardiac hypertrophy (27) and apoptosis and mitochondrial dysfunction in mouse embryonic cells (28).

The effect of increased AEA levels induced by SBFI26 has revealed a gateway for therapeutic potential beyond analgesia. The abuse of prescription and non-prescription opioids, such as heroin, has transitioned from a form of self-medication to a worldwide epidemic (29, 30). Given recent evidence of endocannabinoid modulation of pain and the abuse liability of many opioids, we hypothesize that manipulation of endogenous AEA signaling by SBFI26 might have important clinical potential. It is important first though to establish whether this compound may have abuse liability or exhibit some of the characteristic negative side effects of cannabinoid compounds.

In addition to the well-known “tetrad” of cannabinoid effects [analgesia, hypothermia, catalepsy, and sedation (31)], increased activation of CB1 can lead to aversive reactions and deficits in humans and animals, sometimes in a biphasic manner that contrasts the therapeutic effects of moderate increases in CB1 activation (32). For example, elevation of AEA *via* FAAH administration has produced anxiolytic effects (33), but high doses of CB1 agonists can produce anxiogenic responses (5). Accordingly center time in the open field, which is characterized as a measure of anxiolytic response, is significantly reduced in WT C57 mice administered THC, as is total open field locomotor activity at high doses (34). However, low doses of the synthetic CB1 agonist WIN55,212-2, URB597, and the “AEA transport inhibitor” AM404, which also raises AEA levels, did not decrease open field center time (35).

Learning and memory are also impaired by administration of THC in clinical studies (6, 36, 37) along with a variety of other CB1 agonists in rodents (38, 39). Both URB597 and the stable synthetic AEA analog R-(+)-methanandamide cause deficits in object recognition and performance in a variety of other short-term memory tests in a CB1-dependent manner (40, 41), and it was also recently demonstrated that URB597 impairs LTP in hippocampal brain slices from wild-type, but not CB1^−/−^ mice (40). CB1 receptors are abundant in hippocampus (31), and AEA, but not 2-AG, has been deemed the ligand responsible for CB1-dependent memory deficits when in excess in this brain area (42). Given these findings, it is entirely possible that SBFI26 could potentially cause cognitive and learning deficits.

Similarly, recognition memory for social stimuli is also mediated at least in part by CB1 activity. Selective deletion of CB1 in specific neuronal populations has elicited similar effects on exploration in both the object recognition and social interaction (SI)/social novelty (SN) procedures, and the hippocampus has been primarily implicated in both (43). However, preference for SI and SN is mediated by a range of additional variables, such as olfactory, auditory, and ectocrine cues, as well as unshared neural correlates such as the involvement of the hormones oxytocin and vasopressin (44). In turn, although the tests for SI and SN can be used to operationalize aspects of learning and memory as well as anxiogenic responses (34), these paradigms are reflective of combinations of multiple behaviors, are considered a model for characterizing many different psychiatric disorders, and may reveal treatment effects not observed by testing recognition memory and exploratory drive alone (45).

Perhaps most importantly, pharmacological ECS manipulation has brought up the concern of addictive properties associated with the variety of compounds used to achieve this. Squirrel monkeys, for example, have been shown to self-administer AEA, an effect blocked by CB1 antagonism (46). Conversely, Scherma et al. (47) showed that while mice receiving intravenous injection of anandamide did not induce a conditioned place preference (CPP), the introduction of URB597 along with AEA resulted in conditioned place aversion. More recently, squirrel monkeys were also shown to self-administer AM404, and this compound also reinstated drug seeking for THC and cocaine (48). Similar to THC, it appears AEA might exhibit varying qualities under particular conditions that warrant further explanation. The present study examined the effects of the specific FABP5/7 inhibitor SBFI26 on CPP, a well-established model used to measure the rewarding properties of stimuli. Additionally, we studied a range of other behaviors previously shown to be affected by endocannabinoids in attempt to characterize any cannabimimetic side effects and potential clinical utility.

## Materials and Methods

### Animals

Male C57BL/6 mice (22–30 g, 9–10 weeks old, Taconic Farms) were used for all experiments (*n* = 66). The animals were single housed at room temperature (22°C) and in controlled humidity conditions and kept on a 12-h inverted light cycle beginning at 0900 hours with *ad libitum* access to water and food. Food intake and body weights were monitored daily. The animals were habituated to the experimental room for 1 week before testing. The experiments conducted herein conform to the National Institutes of Health Guidelines for the Care and Use of Laboratory Animals and were approved by the University Institutional Animal Care and Use Committee. Mice were divided into four treatment groups (vehicle, 5.0, 20.0, or 40.0 mg/kg SBFI26).

### Drugs

The FABP inhibitor SBFI26 was synthesized as in Berger et al. (21). The drug was dissolved in DMSO:cremophor-EL:saline (4% DMSO:10% Cremophor-EL) and administered intraperitoneally at a volume of 10 μl/g body weight. Doses used were determined by our previous report with SBFI26 (26).

### Equipment

The CPP apparatus used was a Habitest three-chamber model (Coulbourn Instruments; Allentown, PA, USA), as previously described (49). The present study used 1″ black and white stripes on two opposite walls in the left compartment, which contained crisscrossed half-inch metal flooring, whereas the right compartment consisted of perforated stainless-steel flooring with round holes on staggered centers, and 1″ black and white checkering on two opposite walls.

Locomotor and exploratory behavior were examined using an open field arena and the Tru Scan software photo beam tracking system (Coulbourn Instruments, Whitehall, PA, USA). The open field arena was a 16″ × 16″, and the perimeter was designated as the area within 2.5″ of the walls. The center consisted of the area within the perimeter.

Novel object recognition (NOR) testing also took place in the open field arena, and objects used were similar 5″ pink buckets and white vases. SI and SN testing took place in a plexiglass three-chamber apparatus, as described in Ref. (50), and used 5″ black wire cups to hold stimulus mice. Tests were recorded by digital video camera.

### Procedures

#### Conditioned Place Preference

The CPP paradigm was performed, as described in Ref. (49, 51), with some modifications (60 min conditioning sessions and 15 min pretest/test). SBFI26 or vehicle was administered 50 min prior to each conditioning session, and animals were returned to their home cage between injection and testing. The present study determined preference by calculating the percentage of time spent in one compartment compared to the other. This percentage is regarded as *percent preference*. Compartment preference for CPP was evaluated by comparing the percent time (time in one chamber divided by total time spent in both chambers) spent in the drug-paired chamber on preconditioning day to test day. Locomotor activity during CPP was also measured each conditioning day and binned for a time period of 1 h.

#### Open Field Activity

Locomotor activity and exploratory behavior were measured in an open field arena for 1 h, as previously described (52). Measurements included total distance traveled (FP Distance), time spent in center (Center Time), and time spent in a rearing position (VP Time). SBFI26 injections took place 50 min before the animal entered the open field.

#### Novel Object Recognition

Novel object recognition was carried out, as previously described (53). During the “familiar” phase, mice were placed in the center of the arena and allowed to explore two identical objects in either corner for 5 min. They were then returned to their homecage for 30 min, during which time one of the “familiar” objects was switched with a “novel” object. Subjects were then returned to the arena for the “novel” phase, and the proportion of time spent exploring the novel object over total exploration was scored for analysis. Buckets and vases were counterbalanced, as being considered the “familiar” and “novel” objects to avoid bias. SBFI26 injections occurred 50 min prior to the middle of the “familiar” run. Exploration of an object was considered to be occurring if the test mouse was within 1″ of the circumference of the object and oriented toward it. Recorded videos were scored by two research assistants, and scored were compared with an inter-rater reliability score.

#### Social Interaction and Novelty

Social interaction and SN were performed in a three-chambered arena with open doors, as described in Ref. (50), with minor modifications. Briefly, the test began with a 5-min habituation period in which the entire arena with both empty cups could freely be explored. Water bottles were placed atop cups to prevent climbing. The experimentor then introduced a conspecific male stimulus mouse to one of the cups for a 10-min “familiar” run (SI), followed immediately by the introduction of another conspecific male in the other cup in the “novel” run (SN). After 10 more minutes of exploration, all mice were returned to their home cages. SBFI26 injections occurred 50 min prior to the middle of the “familiar” run. Exploration of a conspecific was considered to be occurring if the test mouse was within 1″ of the circumference of the cup and oriented toward it. Recorded videos were rated with Top Scan software (Clever Sys Inc., VA, USA).

### Statistical Analysis

Paired-samples *t*-tests were used to calculate differences in preference between CPP preconditioning and test day for each treatment group, followed by a one-way ANOVA to detect differences in preference on test day between treatment groups. A two-way repeated measures (RM) ANOVA with SBFI26 dose and treatment (SBFI26 or vehicle) as factors was used to analyze locomotor activity throughout CPP conditioning. Open field parameters, NOR, SI, and SN were compared between treatment groups using one-way ANOVAs. All statistics and graphing were performed using SigmaPlot 11.0 software (Systat Software Inc., San Jose, CA, USA).

## Results

### Conditioned Place Preference

**Figure 1:**
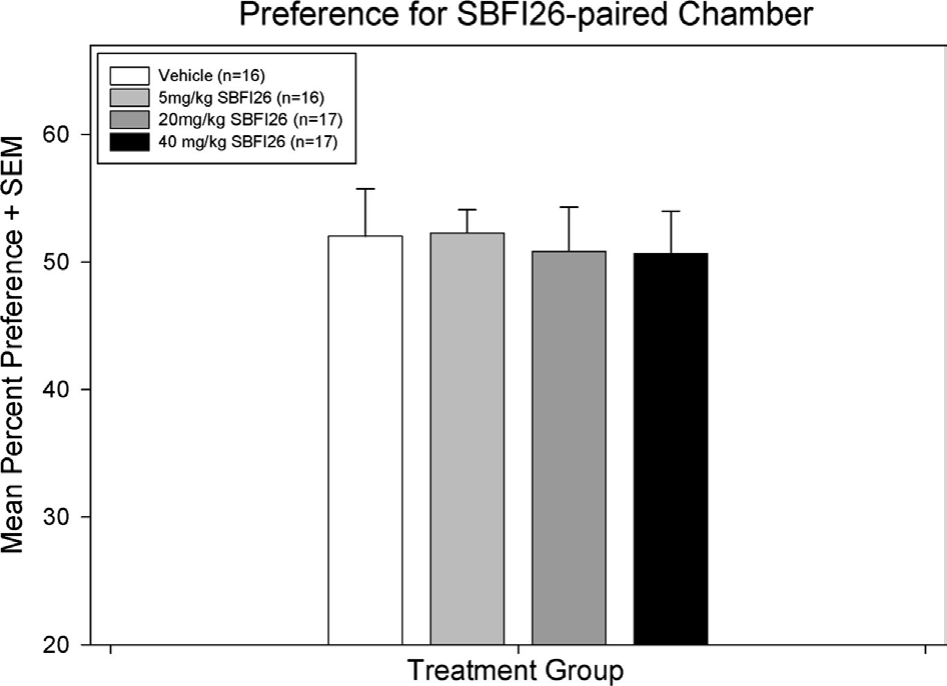
**A paired-samples *t*-test found no significant difference within any treatment group between percent preference for the drug-paired chamber on preconditioning day versus test day**.

Paired-samples *t*-tests found no significant preference for the SBFI26-paired chamber with any of the treatment doses administered (see Figure 1); vehicle [*t*(12) = −1.18, *p* = 0.26], 5.0 mg/kg [*t*(15) = −1.87, *p* = 0.081], 20.0 mg/kg [*t*(15) = −1.79, *p* = 0.094], and 40.0 mg/kg [*t*(14) = −1.87, *p* = 0.082]. There was also no difference between treatment groups in mean preference on test day [*F*(3,56) = 0.060, *p* = 0.98].

### CPP – Locomotor Activity

A two-way RM ANOVA on average total activity between treatment groups and treatment days revealed no significant differences between groups [*F*(3,58) = 1.81, *p* = 0.16] or treatment day [*F*(1,58) = 1.11, *p* = 0.30; figure not shown].

### Open Field

**Figure 2:**
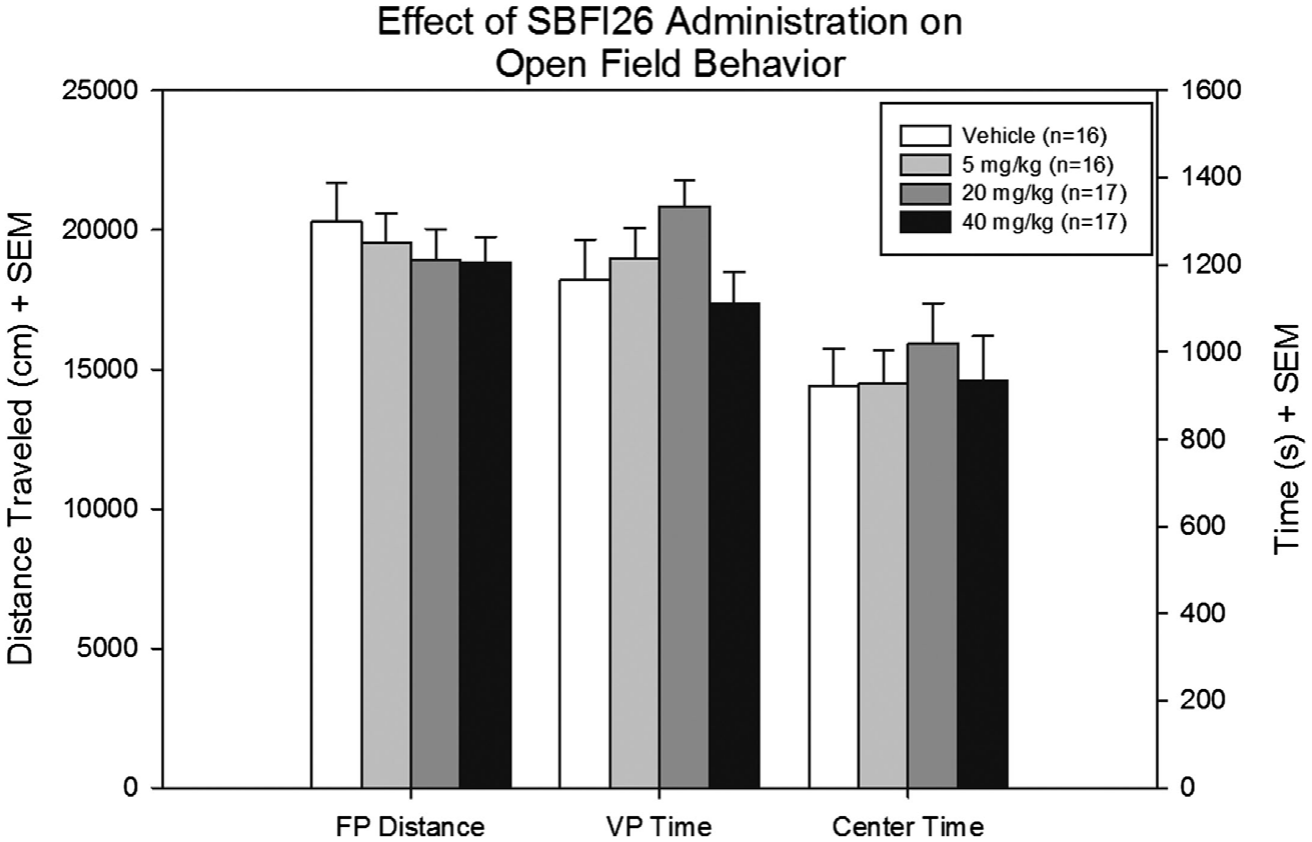
**Floor plane distance traveled, vertical plane time, and center time were measured for all mice treated with either vehicle SBFI26 in the open field test**. No significant difference was found between groups in all three parameters.

No differences were revealed between treatment groups with respect to FP distance, a secondary measure of locomotor activity [*F*(3,53) = 0.36, *p* = 0.78]. There were also no differences in time spent in the vertical plane [*F*(3,53) = 1.63, *p* = 0.19] and time spent in the center [*F*(3,53) = 0.25, *p* = 0.86], and no significant interaction detected (Figure 2).

### Novel Object Recognition

The proportion of time spent exploring the novel object did not differ with respect to SBFI26 dose [*F*(3,53) = 0.31, *p* = 0.82]. A one-way ANOVA also did not fine any significant differences in total object exploration time between treatment groups [*F*(3,53) = 0.78, *p* = 0.51; figure not shown].

### Social Interaction and Novelty

Separate one-way ANOVAs were conducted to analyze the acute effects of SBFI26 on social behavior – defined as SI and SN. No significant main effect was found for the treatment of SBFI26 doses given [*F*(3,38) = 2.07, *p* = 0.12; figure not shown] on SI (interaction time with cup versus mouse). Similarly, no effect was found on SN (interaction time with familiar mouse versus novel mouse) for the SBFI26 doses given [*F*(3,38) = 1.579, *p* = 0.210].

## Discussion

By targeting the intracellular anandamide transporters FABP5 and FABP7, this study examined the effects of SBFI26 on CPP reward behavior, locomotor and exploratory activity in the open field, NOR, SI, and SN. Results showed that SBFI26 did not induce CPP nor aversion. In addition, SBFI26 did not have any effects on locomotor activity measured during CPP conditioning or in the open field. We also found that SBFI26 did not have effects on other behaviors measured.

THC and other CB1 agonists have been shown to exhibit a biphasic effect on reward and locomotion (54) as well as anxiety (55), and elevate dopamine (DA) in the nucleus accumbens such as all known drugs of abuse (56). FAAH inhibitors vary in their ability to produce rewarding effects and increases in DA concentrations in the nucleus accumbens; these effects are specific to which compound is being administered (57), as well as route of administration (58). Therefore, it was unknown if SBFI26 would have rewarding effects. The present study demonstrates that SBFI26 does not elicit the acquisition of reward or CPP behavior or CPA (aversion). Given that we recently reported the analgesic and anti-inflammatory properties of SBFI26 (26), the use of SBFI26 for pain management may provide future promise.

In addition, SBFI26 was shown not to have any effects on multiple test parameters of locomotor and exploratory activity, working memory as measured by NOR, and propensity for SI and SN. Specifically, SBFI26 did not produce differences in activity during the CPP procedure or the open field parameters of distance traveled, time in the center, or exploratory or rearing behavior (as measured by time spent in the vertical plane). Proportion of time exploring the novel object in NOR, and time spent exploring the stimulus mouse in SI and SN also revealed no differences between SBFI26 treatment groups. Due to the previously mentioned biphasic action of cannabinoids, we chose to utilize an effective dose of SBFI26 (20.0 mg/kg) for these experiments, as well as a low and high dose to tease out potential side effects across different degrees of FABP5/7 inhibition. SBFI26 did not cause any behavioral changes in the tests conducted at any dose.

These findings are in contrast to previous studies showing deficits in NOR induced by acute doses of CB1 agonists and R-(+)-methanandamide (41), as well as URB597 (40, 42), and decreased SI time in control mice (34). The latter finding is particularly intreaguing, given recent evidence that oxytocin elevates AEA in the nucleus accumbens to facilitate social reward (59). One possible explanation for the lack of significant differences in behavior in the present study is the degree to which SBFI26 raises AEA levels – URB597 has been shown to cause a fourfold increase of this endocannabinoid in mouse brain (5), whereas our group detected significant but smaller increases from SBFI26 (26). Therefore, SBFI26 may raise AEA concentrations to a degree that can still have therapeutic effects without detriments. Although we did not detect any anxiolytic effects of acute SBFI26 administration as measured by center time in the open field, this realm of research warrants further investigation given previous data of AEA elevation on anxiety (60).

These results are encouraging given the potential analgesic and anti-inflammatory properties of SBFI26, but more research is needed on the effects of SBFI26 testing on other drug abuse paradigms (i.e., self-administration, locomotor sensitization). Future research should also be directed at interactions with commonly abused drugs, such as cocaine, heroin, and marijuana.

## Author Contributions

All authors have significantly contributed to the research described in this article. Developed/planned the experiment: PT, SH-D, DD, and MK; performed the experiments: BC, JO, TM, CK, and EM; analyzed the data: PT, BC, TM, CK, AE, and JL; wrote the paper: PT, BC, and JH.

## Conflict of Interest Statement

The authors declare that the research was conducted in the absence of any commercial or financial relationships that could be construed as a potential conflict of interest.
